# Molecular Breeding for Ascochyta Blight Resistance in Lentil: Current Progress and Future Directions

**DOI:** 10.3389/fpls.2017.01136

**Published:** 2017-06-29

**Authors:** Matthew S. Rodda, Jennifer Davidson, Muhammad Javid, Shimna Sudheesh, Sara Blake, John W. Forster, Sukhjiwan Kaur

**Affiliations:** ^1^Agriculture Victoria, Grains Innovation ParkHorsham, VIC, Australia; ^2^Pulse and Oilseed Pathology, Plant Health and Biosecurity, Sustainable Systems, South Australian Research and Development Institute, UrrbraeAdelaide, SA, Australia; ^3^Agriculture Victoria, AgriBio, Centre for AgriBioscience, La Trobe UniversityBundoora, VIC, Australia; ^4^School of Applied Systems Biology, La Trobe UniversityBundoora, VIC, Australia

**Keywords:** legume, pulse, mapping, molecular markers, fungal disease resistance

## Abstract

Lentil (*Lens culinaris* Medik.) is a diploid (2n = 2x = 14), self-pollinating, cool-season, grain legume that is cultivated worldwide and is highly valuable due to its high protein content. However, lentil production is constrained by many factors including biotic stresses, majority of which are fungal diseases such as ascochyta blight (AB), fusarium wilt, rust, stemphylium blight, anthracnose, and botrytis gray mold. Among various diseases, AB is a major -problem in many lentil-producing countries and can significantly reduce crop production. Breeding for AB resistance has been a priority for breeding programs across the globe and consequently, a number of resistance sources have been identified and extensively exploited. In order to increase the efficiency of combining genes from different genetic backgrounds, molecular genetic tools can be integrated with conventional breeding methods. A range of genetic linkage maps have been generated based on DNA-based markers, and quantitative trait loci (QTLs) for AB resistance have been identified. Molecular markers linked to these QTLs may potentially be used for efficient pyramiding of the AB disease resistance genes. Significant genomic resources have been established to identify and characterize resistance genes, including an integrated genetic map, expressed sequence tag libraries, gene based markers, and draft genome sequences. These resources are already being utilized for lentil crop improvement, to more effectively select for disease resistance, as a case study of the Australian breeding program will show. The combination of genomic resources, effective molecular genetic tools and high resolution phenotyping tools will improve the efficiency of selection for ascochyta blight resistance and accelerate varietal development of global lentil breeding programs.

## Introduction

Lentil is a self-pollinating diploid (2n = 2x = 14) grain legume crop with a genome size of c. 4 Gbp (Arumuganathan and Earle, 1991). Lentil is cultivated globally and is highly valued as an efficient source of dietary protein. The global cool-season grain legume production is largely represented by chickpea (*Cicer arietinum* L.), pea (*Pisum sativum* L.), and cultivated lentil (*Lens culinaris* Medikus ssp. *culinaris*) (Khazaei et al., 2016). Lentil was one of the oldest domesticated grain legumes, derived from a center of origin in the Near East (Zohary, 1999), and the highest levels of contemporary diversity are still located in this region, particularly Turkey, Syria, and Iraq. Lentil cultivation subsequently spread to the Nile valley, Central Asia and the Mediterranean Basin, followed by Pakistan, India, and South America (Cubero, 1981; Khazaei et al., 2016). The crop is currently grown widely throughout the Indian sub-continent, the Middle East, northern Africa, southern Europe, North and South America, Australia, and western Asia (Fikiru et al., 2007; Kaur et al., 2014a). The total (global) lentil production is estimated at 4.4 million metric tons from an estimated 4.2 million hectares, with an average yield of 1,068 kg/ha (FAO, 2015; Kumar et al., 2015). Lentil cultivation in rotation with cereals provides benefits to the cropping systems through biological nitrogen fixation, breaking of disease cycles and effective control of weeds, and significant support for the livelihood of small-scale farmers practicing agriculture in the dryland agricultural ecosystems of South Asia, Sub-Saharan Africa, West Asia, and North Africa (Kumar et al., 2013).

Lentil production is limited by many factors including abiotic stresses such as terminal drought, heat stress, low soil fertility, and various biotic stresses including infection by the pathogens causing ascochyta blight (*Ascochyta lentis* Vassilievsky), fusarium wilt (*Fusarium oxysporum* f.sp. *lentis*), anthracnose (*Colletotrichum truncatum*), stemphylium blight (*Stemphylium botryosum*), rust (*Uromyces viciae-fabae*), botrytis gray mold (*Botrytis cinerea* and *B. fabae*), and white mold (*Sclerotinia sclerotiorum*) (Sharpe et al., 2013; Kumar et al., 2015). Among these diseases, ascochyta blight (AB) is one of the most widespread, being of economic concern in the majority of lentil-producing regions, especially under the mild, wet winter conditions of Mediterranean and maritime climates (Erskine et al., 1994; Ye et al., 2002; Ford et al., 2011). *A. lentis* (teleomorph *Didymella lentis*) is the causal agent of AB of lentil (Kaiser et al., 1997). Symptoms include lesions on stems, leaves, petioles and pods. Plant death is common following seedling infection, while infection of mature plants can lead to significant reduction in yield and seed quality (Morrall and Sheppard, 1981). The foliar infection can cause yield losses of up to 40%, but the loss of economic value due to seed staining and mold may be more than 70%, as it can result in a failure to meet export quality standards (Gossen and Morrall, 1983, 1984; Brouwer et al., 1995). AB can be managed through the application of fungicides, however the most economically viable and environmentally sustainable method of control is the development of disease resistant varieties (Ford et al., 2011).

As a decade may typically be required for release of a commercial variety, development and implementation of new molecular genetics tools will support a transition from conventional to genomics-assisted breeding approaches in order to accelerate the release of improved lentil cultivars. Molecular tools, including marker-assisted selection, have the potential to accelerate and improve the effectiveness of breeding for disease resistance in lentil. For this reason, during the last two decades substantial efforts have been made to understand the genetics and genomics of lentil, including a focus on understanding the genetic basis of resistance to *A. lentis*. Genetic linkage maps of lentil have been constructed based on a range of molecular genetic marker types such as randomly amplified polymorphic DNAs (RAPDs), amplified fragment length polymorphisms (AFLPs), sequence characterized amplified regions (SCARs), resistance gene analogs (RGAs), simple sequence repeats (SSRs), inter-simple sequence repeats (ISSRs), and single nucleotide polymorphism (SNPs) (Eujayl et al., 1998; Rubeena et al., 2003; Tullu et al., 2006; Sharpe et al., 2013; Kaur et al., 2014a; Verma et al., 2015). Through the use of these maps, a number of genomic regions controlling AB resistance have been identified (Ford et al., 1999; Rubeena et al., 2006; Sudheesh et al., 2016).

In addition to lentil, AB is one of the most important diseases of the other cool season food legumes such as field pea, chickpea, and faba bean, although the causal pathogens of AB differ for each crop host. The status of AB as a disease of significant economic concern in each of these crops has led to a large number of QTL studies aimed at identifying the genomic regions associated with AB resistance; in field pea (Timmerman-Vaughan et al., 2002, 2004; Tar'an et al., 2003a; Prioul et al., 2004; Fondevilla et al., 2008, 2011; Jha et al., 2016, 2017), chickpea (Udupa and Baum, 2003; Lichtenzveig et al., 2006; Tar'an et al., 2007; Sabbavarapu et al., 2013), and faba bean (Román et al., 2003; Avila et al., 2004; Kaur et al., 2014b; Atienza et al., 2016). Within Australia breeding germplasm, lentil is the most advanced of these four crop species in the implementation of MAS for AB resistance (pers. comm. Rodda, Agriculture Victoria). Breeding for resistance to AB in field pea is complicated by the co-occurrence of three to four species in the disease complex also known as blackspot (Bretag and Ramsey, 2001; Davidson et al., 2009). In addition, there are limited sources of major gene resistance available in field pea (Kraft et al., 1998; Zhang et al., 2006). AB is one of the most important diseases of faba bean and chickpea and resistance has been a major focus of molecular marker development for these crops in Australia. Unfortunately, in both these species, there have recently been shifts in the pathogen population, overcoming key resistance genes (Kimber et al., 2016; Moore et al., 2016) which have rendered their available markers unusable for the most aggressive forms of the ascochyta blight pathogens.

In this review paper, the progress of and prospects for breeding for ascochyta blight resistance in lentil is discussed, along with potential impact of genomic technologies on future crop improvement.

## The pathogen

*A. lentis* can infect cultivated and wild species of lentil including *L. culinaris* subsp. *orientalis, L. culinaris* subsp. *odemensis, L. ervoides, L. lamottei, L. nigricans*, and *L. tomentosa* (Bayaa et al., 1994; Hernandez-Bello et al., 2006; Tullu et al., 2010). However, the pathogen appears to be host-specific to the *Lens* genus, being unable to cause disease symptoms on other legume crops including chickpea (*C. arietinum*), faba bean (*Vicia faba*), field pea (*P. sativum*), or hairy vetch (*V. villosa*) (Hernandez-Bello et al., 2006; Peever et al., 2007).

A comparison of the related *Ascochyta* pathogens from wild and cultivated legume hosts, including *A. lentis, A. fabae, A. rabiei, A. pinodes, A. pinodella*, and *A. pisi*, has revealed near-identical ribosomal DNA internal transcribed spacer (ITS) regions. In contrast, analyses of protein-coding genes of fungal isolates obtained from the same host species demonstrated clustering even when collections had been made from diverse regions. A co-evolutionary history between the pathogens and their respective hosts is likely to have resulted in the observed host specificity of *Ascochyta* fungi (Peever et al., 2007).

Early morphological studies revealed that *A*. *lentis* could not be separated from *A. fabae* (the causal pathogen of ascochyta blight on faba bean) on the basis of cultural or morphological characteristics, and so the two pathogens were proposed to be synonymized as two special forms i.e., *A. fabae* f. sp. *lentis* and *A. fabae* f. sp. *fabae*, respectively (Gossen et al., 1986). *A*. *lentis* was later confirmed as a species distinct from *A*. *fabae* on the basis of pathology tests, RAPD markers and the results of controlled crosses between complementary mating types of *A. fabae* and *A. lentis* (Kaiser et al., 1997). Notably, the crosses showed the inability of the progeny to produce fertile pseudothecia that induce disease on either host parent (Kaiser et al., 1997). In contrast, progeny from successful matings between *A. lentis* and an Italian isolate from ascochyta-type lesions on grasspea (*Lathyrus sativus* L.) produced a normal culture morphology, demonstrating that these isolates could not be placed into separate taxa. The variant, which is able to infect grasspea but not lentil, has recently been described as *A. lentis* var. *lathyri*, and shows 99–100% sequence identity to the *A. lentis* genome, despite significant morphological differences between conidia of the two variants. The differences in conidial dimensions and host specificity suggest that these variants have arisen from a speciation process (Infantino et al., 2016).

As a heterothallic fungus, *A. lentis* requires two mating types (MAT1-1 and MAT1-2) for sexual reproduction to occur in order to produce the *Didymella* teleomorph (Kaiser et al., 1997; Galloway et al., 2004; Hernandez-Bello et al., 2006). The two mating types are encoded by alternate alleles at a single (*MAT*) locus. PCR amplicons of sizes 450 and 700 bp have been amplified from MAT1-1 and MAT1-2 isolates, respectively (Cherif et al., 2006) although a MAT1-2-specific amplicon at 750 bp has also been consistently amplified (pers. comm. Herdina, SARDI, March 2017). Both mating types have been identified in isolates from Algeria, Canada, Hungary, India, Russia, Spain, USA (Ahmed et al., 1996a), and Australia (Galloway et al., 2004). MAT1-1 is reported to occur more frequently than MAT1-2 in Australia in the *A. lentis* population by a ratio of 2:1 (Nasir, 1998 cited in Skiba and Pang, 2003) and 5:1 in Canada (Ahmed et al., 1996a).

Sexual reproduction between the mating types results in the development and maturation of pseudothecia on infested lentil straw under cool moist conditions (Kaiser, 1997; Galloway et al., 2004). These structures, which have only been observed on straw (Skiba and Pang, 2003), develop within 17 days at 10°C in controlled conditions (Ahmed et al., 1996a). The dome-shaped pseudothecia contain many bitunicate asci each with 8 hyaline, two-celled ascospores (Skiba and Pang, 2003; Galloway et al., 2004). Asexual flask-shaped pycnidia also develop on infested straw and produce conidia (Skiba and Pang, 2003). The maturation of pseudothecia and discharge of ascospores from infested lentil straw overlap with the vegetative stage of the crop (unpublished data, Davidson, SARDI, June 2016), indicating that ascospores may serve as primary inoculum for the disease, similar to the case of *Didymella fabae* (Rubiales and Trapero-Casas, 2002). Mature ascospores of *D. lentis* are wind-dispersed to a distance of 50 m from the infested straw (Galloway and MacLeod, 2002). Epidemics can also be initiated by infested seed (Kaiser and Hannan, 1986) and by asexual conidia which are splash-dispersed from infested straw onto lentil plants during rainfall (Morrall and Sheppard, 1981; Kaiser and Hannan, 1986; Nasir and Bretag, 1997b). Spores can germinate within 6 h of inoculation, and germ tubes grow to form an appressorium within 10 h (Roundhill et al., 1995). Under optimal conditions of temperature (15–20°C) and leaf wetness, the period from inoculation to expression of disease symptoms for *A. lentis* is 6–7 days (Pederson and Morrall, 1994), but may take up to 10–14 days (Roundhill et al., 1995). Necrotic lesions, initially pale green and then turning light brown, develop on all above-ground parts, leading to leaf drop, stem breakage, reduction in pod size, and shriveled and/or stained seed. Pycnidia and conidia develop within the lesions on diseased plants during the growing season, and the epidemic spreads to adjacent plants through successive cycles of rain-splashed conidia (Pederson et al., 1994; Ford et al., 2011).

## Host-pathogen interactions

*A. lentis* populations are highly variable in terms of aggressiveness on different lentil cultivars and wild accessions (Bayaa et al., 1994; Ahmed et al., 1996b; Nasir and Bretag, 1997a; Ahmed and Morrall, 1999; Tullu et al., 2010; Davidson et al., 2016). There was also a greater degree of variability identified in populations of *Ascochyta* spp. isolated from wild host species, suggesting that the collections from cultivated hosts constitute sub-sets of the variation present in wild populations (Peever et al., 2007). Studies using different host sets of *L. culinaris* each identified five or six pathotypes of *A. lentis* in Australia (Nasir and Bretag, 1997a, 1998; Sambasivam et al., 2017) and Pakistan (Iqbal et al., 2006). Pathogenic groups were also separated by differences in pre-penetration events (spore germination, germ tube length, and appressoria development), and early differences in defense responses (Sambasivam et al., 2017).

There is no evidence to suggest that mating type influences the aggressiveness or virulence of pathogen isolates (Ahmed et al., 1996a). However, the presence of both mating types of *A. lentis* leads to a high potential for adaptation through sexual reproduction, since heterothallism ensures a diverse population (Ford et al., 2000; Cherif et al., 2006). In addition, the movement of infected seed between regions, as well as the introduction of isolates via international germplasm (Kaiser, 1997), increases the potential for pathogenic variability and generation of isolates with increased aggressiveness. RAPD analysis revealed greater variability among isolates from Western Australia than those from a larger geographical area in eastern Australia, presumably due to multiple introductions from international sources into Western Australia (Ford et al., 2000).

Intensive cropping of single cultivars can lead to loss of resistance by selection for aggressive isolates that are already present in the naturally variable population (Davidson et al., 2016). Recent changes in the foliar response of the previously resistant lentil cvs. Northfield (ILL5588) and Nipper were identified and experimentally confirmed in Australia (Davidson et al., 2016), the latter being a progeny of the resistant cvs. Northfield x Indianhead. ILL5588 was used extensively as a source of resistance to AB in the Canadian and Australian lentil breeding programs (Tullu et al., 2010; Davidson et al., 2016), and these changes may have a wide impact on resistant sources. There was also loss of resistance to AB in Canada on lentil cv. Laird, leading to 50% yield reduction (Morrall, 1997). Rapid loss of resistance to AB indicates resistance conferred by major genes, but the general continuum of aggressiveness that is also present among *A. lentis* isolates is indicative of polygenic resistance, leading to the conclusion that both major and minor genes are involved (Ye et al., 2002; Banniza and Vandenberg, 2006; Gupta et al., 2012; Davidson et al., 2016).

Inheritance of pathogen virulence on cv Northfield (ILL5588) was reported to be controlled by two independently segregating genes, operating in mutual epistasis, based on a 3:1 segregation ratio in the F_1_ progeny (ascospores) (Skiba and Pang, 2003). Because *A. lentis* is a haploid organism, the F_1_ progeny between virulent and avirulent isolates should segregate, while two virulent isolates should only produce virulent progeny. Ahmed and Morrall (1999) identified avirulent progeny from crosses between parents of intermediate virulence and also crosses between two highly virulent parents. Some progeny of each cross showed intermediate reactions as compared to the parents. These results may indicate the involvement of multiple genes with additive effects, and/or gene interaction. In addition, some progeny displayed higher virulence than either parent, showing that sexual recombination can generate novel isolates capable of attacking AB resistant cultivars.

In order to begin to understand the complexity of genes involved in resistance to *A. lentis*, a micro-array experiment with 762 probes was used to investigate gene expression changes in the susceptible lentil line ILL6002 and the resistant line ILL7537 (Mustafa et al., 2009). Several differentially expressed genes encoding pathogenesis-related (PR) proteins were identified in the early stages of infection, including a PR4 protein, three PR10 proteins and a β-1,3-glucanase, all up-regulated in the resistant ILL7537 line but not the susceptible ILL6002 line. β-1,3-glucanases cause lysis of the fungal cell wall, while PR4 disrupts cell growth through chitinase activity. Both mechanisms may work in tandem: the first opening the cell wall, so allowing the second to enter the cell and disrupt function. The pathogenesis-related PR4-encoding gene *LcPR4a* (Vaghefi et al., 2013), which was induced in lentil plants following infection by *A. lentis*, was detected at 12 h post-infection in both compatible and incompatible interactions of plant and pathogen. However, the magnitude of *LcPR4a* expression continued to increase in the resistant line to 114-fold by 48 h post-infection. Recombinant *LcPR4a* protein significantly reduced fungal biomass in an *in vitro* antifungal assay, further suggesting a role in the defense response to *A. lentis* (Vaghefi et al., 2013).

## Genetics of AB resistance

Several studies have been performed to explore the genetics of both seed/pod and foliar AB resistance in lentil, with resistant germplasm identified in both the cultivated and wild species (Bayaa et al., 1994; Tullu et al., 2010). An early study identified foliar AB resistance in wild lentil species, controlled by two dominant genes in both *L. ervoides* (Brign.) Grande and *L. odemensis* Ladz., and by a single dominant gene in *L. culinaris* ssp. *orientalis* (Ahmad et al., 1997). Several studies have described the roles of both dominant and the recessive genes in conferring AB resistance in cultivated lentil. For example, two foliar AB resistance genes, designated *Ral2* (dominant) and *ral2* (recessive), were identified as being present in the cultivars Northfield and Indianhead, respectively (Andrahennadi, 1994; Chowdhury et al., 2001). In addition, a third distinct dominant gene for foliar resistance (*AbR*_1_) has been reported from Northfield (Tay and Slinkard, 1989). Two dominant complementary genes have been found to be associated with inheritance of foliar AB resistance in lentil accession ILL7537 (Nguyen et al., 2001). Previous reports indicated that screening of this accession with molecular genetic markers linked to, and flanking, the resistance gene *AbR*_1_ failed to identify the resistance marker alleles, indicating that the AB resistance in ILL7537 may potentially be unique (Nguyen et al., 2001; Rubeena et al., 2006).

Both dominant and recessive genes were reported to control the seed-based AB resistance in lentil. For example, in one study, a three-gene model for seed-based AB resistance was proposed, including the effects of two dominant genes and a single recessive gene (Tay, 1989). In contrast, another study reported only one dominant and one recessive gene for seed-based AB disease resistance (Sakr, 1994), and a third study reported control by a single dominant gene (Vakulabharanam et al., 1997).

The studies conducted to date on both foliar and seed-based AB resistance have provided a detailed understanding of the role of dominant and recessive genes. The variable number and nature of genes observed in such studies was often due to the different sources of genetic resistance used, with their independent genetic control of plant resistance. In addition, there may be due to differences in AB screening assays, environmental conditions, *A. lentis* isolates and the variable size of populations being evaluated (Ford et al., 1999).

Wild species have the potential to be an important source of resistance to biotic stresses in lentil, compensating for the comparatively low intraspecific variability that is characteristic of domesticated lentil species (Abo-elwafa et al., 1995; Tullu et al., 2010). Interspecific crosses and populations are already being exploited by lentil breeders to introgress diverse resistance genes for a number of other biotic stresses (pers. comm. Vandenberg, University of Saskatchewan).

## Marker-assisted breeding for ascochyta blight resistance

Marker-assisted selection (MAS) allows the selection of a desirable trait with a marker, or suite of markers, based on associated sequence variation, in the absence of direct phenotypic assessment. This approach is dependent on establishment of a close linkage between the molecular genetic marker and the chromosomal location of the gene(s) that control the trait that is to be selected in a particular environment. For example, disease resistance can be evaluated using MAS in the absence of infection, and in the early stages of plant development.

In the major crop species, a large number of genetic markers for key traits relevant to plant breeding are available, providing a critical tool to increase selection efficiency (Dwivedi et al., 2007; Xu and Crouch, 2008). Although application of MAS to lentil has been limited until recently (Kumar et al., 2015), the advent of next-generation sequencing (NGS) technologies provided opportunities for the development of DNA sequence-based markers, which are being implemented in the modern lentil breeding programs of Australia and Canada (pers. comm. Vandenberg, University of Saskatchewan; pers. comm. Rodda, Agriculture Victoria).

A broad range of genetic and genomic resources have recently been generated for lentil through delivery of large numbers of expressed sequence tag (EST)-derived (and hence gene-associated) SSR and SNP markers (Kaur et al., 2011; Sharpe et al., 2013; Kaur et al., 2014a; Sudheesh et al., 2016). They have been extensively used to construct densely populated intraspecific genetic linkage maps, and to identify QTLs (Sharpe et al., 2013; Kaur et al., 2014a; Sudheesh et al., 2016). The information from multiple population-specific genetic maps can be integrated to produce high-density consensus structures utilizing the sequence-linked genetic markers which enables the identification of bridging loci between maps (Sudheesh et al., 2015a,b).

In lentil, molecular markers have been developed for traits with both simple (qualitative) and complex (quantitative) control. In the case of traits controlled by major genes, relatively simple phenotyping methods have been developed, allowing the accurate mapping of the gene. Traits such as boron toxicity tolerance are predominantly controlled by single genes, permitting deployment of a small set of flanking markers (Kaur et al., 2014a). However, to establish reliable marker-trait associations for more complex traits, rapid and reliable screening methods, together with marker saturated target regions and validated QTLs in multiple environments and genetic backgrounds are essential.

A number of independent studies (summarized in Table 1) have used molecular genetic marker technology to determine the basis for AB resistance, based on the construction of genetic maps for segregating populations derived from crossing of parental genotypes with divergent phenotypes. Several generations of marker technology have been used, from low-fidelity and non-locus-specific systems such as RAPDs, AFLPs, and ISSRs (Ford et al., 1999; Chowdhury et al., 2001; Tar'an et al., 2003b; Rubeena et al., 2006; Tullu et al., 2006) to high-fidelity, locus-specific and frequently gene-associated systems such as RFLPs, SSRs, and SNPs (Gupta et al., 2012; Sudheesh et al., 2016). Cultivar Northfield (ILL5588) has been a common parent in the majority of the published studies. Evaluation of resistance has generally been performed at the seedling stage, 11–28 days after infection, although Gupta et al. (2012) co-assessed resistance in both the seedling and mature pod-bearing plant. Most studies revealed multiple QTLs for AB resistance, with magnitude varying from 3 to 89% of the phenotypic variance (V_p_). Due to differing nomenclature systems for linkage groups (LGs) and a dearth of common marker loci between genetic maps, common QTL locations between studies are difficult to establish. Nonetheless, AB_NF1 on LG6 in the study of Sudheesh et al. (2016) is comparable in position to QTL5 on LG1 of Rubeena et al. (2006) and QTL1 on LG1 of Gupta et al. (2012), based on a common SSR locus location. QTLs have also been correlated with known resistance determinants such the dominant *Ral2* and *AbR*_1_ and recessive *ral2* genes (Ford et al., 1999; Chowdhury et al., 2001; Tar'an et al., 2003b).

**Table 1:** Details of the genetic linkage maps and linked markers for ascochyta blight resistance in lentil.

**Name of population**	Assessment tissue–period (DAI^*^)	**QTL name**	**Chromosome/ linkage group**	**Marker type**	**Markers associated with QTL**	**Phenotypic variation explained (%)**	**References**
ILL5588 (cv. Northfield) x ILL6002	Seedling-11	–	–	RAPD	RV01–RB18	89	Ford et al., 1999
ILL5588 (cv. Northfield) x L692-16-1	Seedling-14	QTL 1	LG 2	RAPD, ISSR, RFLP, AFLP	OPB18_680_	36	Tar'anr et al., 2002
		QTL 2	LG 4		OPV1_800_	29	
ILL5588 (cv. Northfield) x ILL7537	Seedling-14	QTL-1	LG2	RAPD, ISSR, AFLP	W03_1050_–S01_750_	11	Rubeena et al., 2006
	Seedling-21	QTL-2	LG2		G04_530_–AC02_480_	7	
		QTL-3	LG4		T16_500_–C04_580_	7	
		QTL-4	LG5		U14_560_–B08_520_	69	
		QTL-5	LG1		B18_1100_–W08_800_	55	
	Seedling-28	QTL-2	LG2		P08_1200_–G04_530_	9	
		QTL-4	LG5		U14_560_–B08_520_	52	
		QTL-5	LG1		B18_1100_–W08_800_		
ILL7537 x ILL6002	Seedling-14	QTL-6	LGI	RAPD, ISSR, AFLP	C-CTA/M-ACC_190_–C-TTA/M-AC_285_	8	Rubeena et al., 2006
		QTL-7	LGI		C-TTA/M-AC_285_–C-TTA/M-AC_165_	27	
		QTL-8	LGII		M20_700_–C-GTA/M-GC_191_	6	
	Seedling-21	QTL-6	LGI		C-CTA/M-ACC_190_–C-TTA/M-AC_285_	11	
		QTL-7	LGI		C-TTA/M-AC_285_–C-TTA/M-AC_165_	34	
		QTL-8	LGII		M20_700_–C-GTA/M-GC_191_	9	
	Seedling-28	QTL-6	LGI		C-CTA/M-ACC_190_–C-TTA/M-AC_285_	16	
		QTL-7	LGI		C-TTA/M-AC_285_–C-TTA/M-AC_165_	31	
		QTL-8	LGII		M20_700_–C-GTA/M-GC_191_	10	
Eston x PI 320937	Seedling-10	QTL	LG-6	RAPD, AFLP, SSR	cagaggE	41	Tullu et al., 2006
ILL5588 (cv. Northfield) x ILL5722 (cv. Digger)	Seedling-14	QTL 1	LG1	EST-SSR/SSR, ISSR, RAPD, ITAP	DK 225–UBC825c	6	Gupta et al., 2012
		QTL 3	LG9		UBC890–ARG10	3	
	Seedling-21	QTL 2	LG1		AC097a–V20a	8	
		QTL 3	LG9		UBC890–ARG10	6	
	Seedling-28	QTL 2	LG1		AC097a–V20a	7	
		QTL 3	LG9		UBC890–ARG10	4	
							
	Pod/maturity-14	QTL 4	LG1		ILMs25–UBC857b	7	
		QTL 5	LG4		UBC855a–UBC830b	7	
		QTL 6	LG5		UBC807a–Lup91	7	
	Pod/maturity-21	QTL 4	LG1		ILMs25–UBC857b	8	
		QTL 5	LG4		UBC855a–UBC830b	7	
		QTL 6	LG5		UBC807a–Lup91	6	
	Pod/maturity-28	QTL 4	LG1		ILMs25–UBC857b	6	
		QTL 5	LG4		UBC855a–UBC830b	7	
		QTL 6	LG5		UBC807a–Lup91	6	
Indianhead x Northfield	Seedling-14	AB_IH1	LG2	Genomic DNA-derived SSR, –EST-SSR, SNP	PBA_LC_0629–SNP_20005010	47	Sudheesh et al., 2016
		AB_IH1.2	LG3		SNP_20002370–SNP_20002371	15	
		AB_NF1	LG6		SNP_20001370–SNP_20001765	7	
Indianhead x Digger	Seedling-14	AB_IH1	LG2	Genomic DNA-derived SSR, –EST-SSR, SNP	SNP_20005010–SNP_20004695	30	Sudheesh et al., 2016
		AB_IH1.3	LG3		SNP_20000505–SNP_20000553	22	

* *DAI, Days after inoculation*.

Although the full genetic basis of AB resistance is not known, screening of a range of lentil genotypes against differential *A. lentis* isolates has identified putative groupings of genotypes based on resistance profiles (Davidson et al., 2016). Molecular genetic marker studies have identified three trait-linked markers relevant to Australian breeding germplasm (Sudheesh et al., 2016). One of these markers, AB_IH1 (see Table 1), is linked to a key resistance gene, which predicted field AB resistance in more than 85% of diverse validation panel, composed both Australian and international germplasm. The currently described AB resistance-associated markers permit selection of two major resistance genes of importance, one from Indianhead and one from ILL5588. However, other lentil germplasm displays resistance to AB that is not explained by these resistance genes, implying that there are additional important resistance genes still to be located in the genome.

## Progress in breeding for AB resistance: an australian case study

The Australian lentil cropping zone is located predominantly in regions of mild, wet winters, in which conditions conducive to fungal disease occur in most years. For this reason, resistance to AB has been considered a priority since the crop was first introduced to Australia, with a significant amount of research and breeding effort put into accessing and introgressing sources of resistance to *A. lentis*.

Given the similarities of climate, the Australian lentil breeding program was based on germplasm developed at ICARDA in Syria, which has been found to be the most readily adapted to Australian conditions. Indeed, many of the early lentil varieties were direct introductions from the ICARDA breeding program, namely cvs. Northfield (ILL5588), Nugget (ILL7180), Digger (ILL5722), Aldinga (ILL5750), and Cumra (ILL0590). Traits for appropriate phenology, yield potential, red lentil seed quality, as well as one source of aschochyta resistance (ILL5588) have been derived from germplasm originating in the Near East. Traits for early vigor and improved biomass were introduced from green lentil germplasm, via North American germplasm, such as cv. Palouse. In terms of disease resistance, the Australian lentil breeding program has benefitted directly from research on AB conducted at the University of Saskatchewan, having utilized AB resistance genes obtained from cv. Indianhead and one of its progeny, cv. CDC Matador.

In the 25 years of lentil breeding in Australia, the program has successfully combined multiple sources, both major and minor, of resistance to AB. These have included the unique resistance sources of ILL5588, Indianhead and potentially another unidentified resistance source (represented by cv. PBA Jumbo2), as well partial (minor) resistance genes, such as those from cv. Digger. To achieve this outcome, the program has relied heavily on field selection for resistance within breeding germplasm, using simple selection methods such as spreading of naturally infected crop residues at sites with reliably cool, wet winters, such as at Horsham, Victoria. Phenotypic selection for resistance has been routinely performed on a whole plot basis, so selecting within families to maintain key resistance genes in breeding germplasm.

The result of this breeding effort has been to obtain a high incidence of resistance to current AB pathogen populations in Australian lentil germplasm. This resistance is also robust, and there are multiple lentil cultivars, such as PBA Ace and PBA Jumbo2, which are effectively immune to the dominant AB isolates when tested in the field or under highly controlled conditions.

Under the disease pressure conditions of the southern Australian cropping environment, intensive lentil production has more recently led to the selection of aggressive pathogen isolates that are able to overcome the major resistance genes which are found in dominant cultivars. As previously described, this has resulted in the loss of effectiveness of the important resistance gene derived from ILL5588 (Davidson et al., 2016). Subsequently, in 2016, pathologists found evidence of isolates able to overcome the key resistance gene linked to AB_IH1 (unpublished data, Blake and Davidson, SARDI, March 2017). This gene, derived from cv. Indianhead, has been widely incorporated in both the Australian and Canadian breeding programs, and the finding identifies a significant threat to the AB resistance of current lentil germplasm. A significant change in the pathogen population could render a substantial proportion of existing Australian breeding material more susceptible. For this reason, beginning in 2016, a pre-emptive breeding strategy based on controlled environment selection was initiated to address the challenge. The other ramification of a potential change is that unless new genetic markers are identified, the Australian breeding program may soon be without a means to effectively predict field-based AB resistance.

For several years, the Australia lentil breeding program has been investing in crosses to incorporate additional unique resistance sources, including ILL7537, which has not been extensively exploited within program. However, field-based selection for new resistance genes is not possible while the current genes are effective. New molecular genetic markers would address this problem, and current research is underway to address the issue. In the meantime, as research effort has applied to study of pathogen diversity in Australia (Davidson et al., 2016), differential sets of isolates offer a good opportunity for phenotypic selection.

## Future prospects for resistance breeding in the genomic era

Recent significant advancements in genomic technologies have opened up new opportunities and enabled new strategies in crop breeding. The genome sequences from model and non-model legumes such as *Medicago truncatula* Gaertn., *Lotus japonicas* L., soybean and chickpea are available in the public domain, and so may be used for comparative genomics analysis. These model plant species permit better understanding of plant development, responses to biotic stresses, and evolution. Conserved synteny between the genomes of legume species has been investigated over the last 20 years, as revealed by comparisons of both genetic maps and fully sequenced genomes (Gujaria-Verma et al., 2014). An international sequencing effort is currently underway with the goal to deliver a reference lentil genome, leading to the recent release of an initial draft genome assembly from the cultivar CDC Redberry (Bett et al., 2016). However, this draft genome sequencing information has limited usage with minimal gene annotation and restricted access (http://knowpulse.usask.ca/portal/). Availability of an improved and well annotated lentil genome assembly in future will allow the identification of diagnostic markers for ascochyta blight resistance and assist breeders to track the trait more effectively. This will eventually improve the rate of selection for ascochyta blight resistance and accelerate the rate of varietal development.

Trait dissection for AB resistance has been exclusively based on the use of biparental genetic mapping populations. However, this approach is a laborious and resource-intensive way to identify marker-trait associations from multiple germplasm sources, including ecotypes and land-races. The availability of large numbers of genome-wide distributed SNP markers, especially following completion of the current effort to determine the lentil genome sequence, will permit implementation of genome-wide association mapping studies (GWASs) (Huang and Han, 2014), based on analysis of customized germplasm collections. The resolution of such studies is typically higher than for linkage mapping, permitting discovery of more closely associated genetic markers. The identification of such sequence polymorphism to physical locations within the genome, either through comparative genomics with model legume species such as *M. truncatula*, or on the draft lentil genome sequence directly, will support prediction of candidate genes for AB resistance. Such genes may include resistance (R) genes involved in pathogen race-specific interactions, such as the nucleotide binding site—leucine-rich repeat (NBS-LRR) class, or more generic defense response genes such as chitinases and glucanases. Given identification of such candidate genes, direct modification through the use of genetic transformation or gene editing may be used to verify identity and potentially transfer specific resistance genes into recipient varieties, in order to accelerate the breeding process. Such approaches, however, will require highly efficient plant transformation and regeneration processes for lentil (Akcay et al., 2009). Densely distributed genome-wide markers will also support the use of genomic selection strategies (Meuwissen et al., 2001; Newell and Jannink, 2014), in which the genetic merits of individual genotypes within a breeding program are predicted on the basis of a genomic estimated breeding value (GEBV) derived from the summation of contributory gene effects across the genome.

## Conclusions

AB, caused by *A. lentis*, is an important disease of lentil throughout the world, causing serious yield losses of up to 70% in extreme cases (Gossen and Morrall, 1983). The most efficient means to control this disease is to breed for host resistance without the need for additional inputs. Extensive searches for AB resistance in lentil have been conducted through screening of germplasm, including cultivated varieties, landraces, and closely related species. To accelerate the process of introgressing AB resistance genes into elite backgrounds, molecular genetic tools can be combined with conventional breeding approaches. Molecular markers associated with AB resistance QTLs have been positioned on linkage maps, and these markers can be used for efficient pyramiding of the disease resistance genes.

Significant achievements have been made in lentil genomics to detect important genes that are involved in AB resistance. Valuable resources, such as an integrated genetic linkage map, EST libraries, gene based markers, and draft genome sequences have been generated. The comparative genomics approaches enabled the identification of candidate genes, however, they have not yet been used directly to improve lentil cultivars in the field, but it is highly likely that these approaches will be more commonly used in near future. The availability of large numbers of molecular genetic markers in lentil will also allow the implementation of GWAS and genomic selection approaches. This will further assist in the identification of more closely linked markers for AB resistance in lentil that can be effectively used in breeding. Genomic selection methods will be useful to calculate prediction values for AB resistance in different sets of germplasm and help to trace inheritance of the trait in future generations.

## Author contributions

All authors contributed to the manuscript text. SK prepared the sections on introduction, molecular markers, and genomics. MR wrote the section on breeding aspects. JD and SB drafted the pathology section. SS contributed to the molecular markers and marker assisted selection. MJ drafted the section on genetics basis for resistance and JF prepared the section on future prospects as well as edited the article. All authors read and approved the manuscript.

### Conflict of interest statement

The authors declare that the research was conducted in the absence of any commercial or financial relationships that could be construed as a potential conflict of interest.

## References

[B1] Abo-elwafa A. Murai K. Shimada T. (1995). Intra- and inter-specific variations in *Lens* revealed by RAPD markers. Theor. Appl. Genet. 90, 335–340. 10.1007/BF00221974 24173922

[B2] Ahmad M. Russell A. McNeil D. (1997). Identification and genetic characterization of different resistance sources to ascochyta blight within the genus *Lens*. Euphytica 97, 311–315. 10.1023/A:1003095423132

[B3] Ahmed S. Morrall R. A. A. (1999). Inheritance of virulence in *Ascochyta lentis* of lentil. Lens Newslett. 26, 25–28.

[B4] Ahmed S. Morrall R. A. A. Kaiser W. J. (1996a). Distribution of mating types of *Ascochyta fabae* f. sp. lentis. Can. J. Plant Pathol. 18, 372–353. 10.1080/07060669609500587

[B5] Ahmed S. Morrall R. A. A. Sheard J. W. (1996b). Virulence of *Ascochyta fabae* f. sp. lentis of lentils from Syria. Can. J. Plant Pathol. 18, 354–361. 10.1080/07060669609500588

[B6] Akcay U. C. Mahmoudian M. Kamci H. Yucel M. Oktem H. A. (2009). *Agrobacterium tumefaciens*-mediated genetic transformation of a recalcitrant grain legume, lentil (*Lens culinaris* Medik.). Plant Cell Rep. 28, 407–417. 10.1007/s00299-008-0652-4 19083242

[B7] Andrahennadi C. (1994). Genetics and Linkage of Isozyme Markers and Resistance to Seedborne Ascochyta Infection in Lentil. M. Sc. Thesis, Department of Crop Science and Plant Physiology; University of Saskatchewan.

[B8] Arumuganathan K. Earle E. D. (1991). Nuclear DNA content of some important plant species. Plant Mol. Biol. Rep. 9, 208–218. 10.1007/BF02672069

[B9] Atienza S. G. Palomino C. Gutiérrez N. Alfaro C. M. Rubiales D. Torres A. M. (2016). QTLs for ascochyta blight resistance in faba bean (*Vicia faba* L.): validation in field and controlled conditions. Crop Pasture Sci. 67, 216–224. 10.1071/cp15227

[B10] Avila C. M. Satovic Z. Sillero J. C. Rubiales D. Moreno M. T. Torres A. M. (2004). Isolate and organ-specific QTLs for ascochyta blight resistance in faba bean (*Vicia faba* L). Theor. Appl. Genet. 108, 1071–1078. 10.1007/s00122-003-1514-7 15067393

[B11] Banniza S. Vandenberg A. (2006). Investigations into the population structure of *Ascochyta lentis* in western Canada, in Proceedings 1st International Ascochyta Workshop on grain Legumes, (Le Tronchet).

[B12] Bayaa B. Erskine W. Hamdi A. (1994). Response of wild lentil to *Ascochyta fabae* f. sp.lentis from Syria. Genet. Resour. Crop Evol. 41, 61–65. 10.1007/BF00053049

[B13] Bett K. Ramsay L. Chan C. Sharpe A. Cook D. Penmetsa R. V. (2016). Lentil v1.0 and beyond, in Proceedings of XXIV Plant and Animal Genome Conference, (San Diego, CA).

[B14] Bretag T. W. Ramsey M. D. (2001). Ascochyta spp, in Compendium of Pea Diseases and Pests, 2nd Edn,eds Kraft J. M. Pfleger F. L. (St. Paul, MI: American Phytopathological Society Press), 24–27.

[B15] Brouwer J. B. Bretag T. W. Materne M. A. (1995). Coordinated improvement program for Australian lentils, in 2nd European Conference on Grain Legumes, (Copenhagen), 25.

[B16] Cherif M. Chilvers M. L. Akamatsu H. Peever T. L. Kaiser W. J. (2006). Cloning of the mating type locus from *Ascochyta lentis* (teleomorph: *Didymella lentis*) and development of a mulitplex PCR mating assay for *Ascochyta* species. Curr. Genet. 50, 203–215. 10.1007/s00294-006-0085-y 16847660

[B17] Chowdhury M. A. Andrahennadi C. P. Slinkard A. E. Vandenberg A. (2001). RAPD and SCAR markers for resistance to acochyta blight in lentil. Euphytica 118, 331–337. 10.1023/A:1017581817201

[B18] Cubero J. I. (1981). Origin, taxonomy and domestication, in Lentils, eds Webb C. Hawtin G. (London: Commonwealth Agricultural Bureaux), 15–38.

[B19] Davidson J. A. Hartley D. Priest M. Herdina M. K. McKay A. Scott E. S. (2009). A new species of Phoma causes ascochyta blight symptoms on field peas (*Pisum sativum*) in South Australia. Mycologia 101, 120–128. 10.3852/07-199 19271674

[B20] Davidson J. Smetham G. Russ M. H. McMurray L. Rodda M. Krysinska-Kaczmarek M.. (2016). Changes in aggressiveness of the *Ascochyta lentis* population in southern Australia. Front. Plant. Sci. 7:393. 10.3389/fpls.2016.00393 27065073 PMC4814486

[B21] Dwivedi S. L. Crouch J. H. Mackill D. J. Xu Y. Blair M. W. Ragot M. (2007). The molecularization of public sector crop breeding: progress, problems and prospects. Adv. Agron. 95, 163–318. 10.1016/S0065-2113(07)95003-8

[B22] Erskine W. Tufail M. Russell A. Taygi M. C. Rahman M. M. Saxena M. C. (1994). Current and future strategies in breeding lentil for resistance to biotic and abiotic stresses. Euphytica 73, 127–135. 10.1007/BF00027189

[B23] Eujayl I. Baum M. Powell W. Erskine W. Pehu E. (1998). A genetic linkage map of lentil (*Lens* sp.) based on RAPD and AFLP markers using recombinant inbred lines. Theor. Appl. Genet. 97, 83–89. 10.1007/s001220050869

[B24] FAO (2015). Production Crops. FAOSTAT Statistics Database. Food and Agriculture Organization of the United Nations; Statistics Division, Rome. Available online at: http://www.fao.org/faostat/en/

[B25] Fikiru E. Tesfaye K. Bekele E. (2007). Genetic diversity and population structure of Ethiopian lentil (*Lens culinaris* Medikus) landraces as revealed by ISSR marker. Afr. J. Biotechnol. 6, 1460–1468.

[B26] Fondevilla S. Almeida N. F. Satovic Z. Rubiales D. Patto M. C. V. Cubero J. I. (2011). Identification of common genomic regions controlling resistance to *Mycosphaerella pinodes*, earliness and architectural traits in different pea genetic backgrounds. Euphytica 182, 43–52. 10.1007/s10681-011-0460-8

[B27] Fondevilla S. Satovic Z. Rubiales D. Moreno M. T. Torres A. M. (2008). Mapping of quantitative trait loci for resistance to *Ascochyta pinodes* in *Pisum sativum* subsp. syriacum. Mol. Breed. 21, 439–454. 10.1007/s11032-007-9144-4

[B28] Ford R. Garnier-Géré P. H. Nasir M. Taylor P. W. J. (2000). Structure of *Ascochyta lentis* in Australia revealed with random amplified polymorphic DNA (RAPD) markers. Australas. Plant Pathol. 29, 36–45. 10.1071/AP00006

[B29] Ford R. Inder P. Taylor P. W. J. Lindbeck K. (2011). Ascochyta blight of lentil, in Compendium of Chickpea and Lentil Diseases and Pests, eds Chen W. Sharma H. C. Muehlbauer F. J. (St. Paul, MI: APS Press), 40–42.

[B30] Ford R. Pang E. C. K. Taylor P. W. J. (1999). Genetics of resistance to ascochyta blight (*Ascochyta lentis*) of lentil and the identification of closely linked RAPD markers. Theor. Appl. Genet. 98, 93–98. 10.1007/s001220051044

[B31] Galloway J. MacLeod W. J. (2002). Epidemiology of ascochyta and botrytis diseases, in Crop Updates: Pulse Research and Industry Development in Western Australia, eds Regan K. White P. (Agriculture, Western Australia; Government of Western Australia), 91–95.

[B32] Galloway J. Macleod W. J. Lindbeck K. (2004). Formation of *Didymella lentis*, the teleomorph of *Ascochyta lentis*, on lentil stubble in the field in Victoria and Western Australia. Australas. Plant. Pathol. 33, 449–450. 10.1071/AP04033

[B33] Gossen B. D. Morrall R. A. A. (1983). Effects of ascochyta blight on seed yield and quality in lentils. Can. J. Plant Pathol. 5, 168–173. 10.1080/07060668309501620

[B34] Gossen B. D. Morrall R. A. A. (1984). Seed quality loss at harvest due to ascochyta blight of lentil. Can. J. Plant Pathol. 6, 233–237. 10.1080/07060668409501559

[B35] Gossen B. D. Sheard J. W. Beauchamp C. J. Morrall R. A. A. (1986). *Ascochyta lentis* renamed *Ascochyta fabae* f. sp. lentis. Can. J. Plant Pathol. 8, 154–160. 10.1080/07060668609501820

[B36] Gujaria-Verma N. Vail S. L. Carrasquilla-Garcia N. Penmetsa R. V. Cook D. R. Farmer A. D.. (2014). Genetic mapping of legume orthologs reveals high conservation of synteny between lentil species and the sequenced genomes of Medicago and chickpea. Front. Plant Sci. 5:676. 10.3389/fpls.2014.00676 25538716 PMC4256995

[B37] Gupta D. Taylor P. W. J. Inder P. Phan H. T. T. Ellwood S. R. Mathur P. N. (2012). Integration of EST-SSR markers of *Medicago truncatula* into intraspecific linkage map of lentil and identification of QTL conferring resistance to ascochyta blight at seedling and pod stages. Mol. Breed. 30, 429–439. 10.1007/s11032-011-9634-2

[B38] Hernandez-Bello M. A. Chilvers M. I. Akamatsu H. Peever T. L. (2006). Host specificity of *Ascochyta* spp. infecting legumes of the *Viciae* and *Cicerae* tribes and pathogenicity of an interspecific hybrid. Phytopathology 96, 1148–1156. 10.1094/PHYTO-96-1148 18943504

[B39] Huang X. Han B. (2014). Natural variations and genome-wide association studies in crop plants. Ann. Rev. Plant Biol. 65, 531–551. 10.1146/annurev-arplant-050213-035715 24274033

[B40] Infantino A. Zaccardelli M. Costa C. Ozkilinc H. Habibi A. Peever T. (2016). A new disease of grasspea (*Lathyrus sativus*) caused by *Ascochyta lentis* var. lathyri. J. Plant Pathol. 98, 541–548. 10.4454/JPP.V98I3.008

[B41] Iqbal S. M. Rauf C. A. Ali S. (2006). Pathogenic variability among the isolates of *Ascochyta lentis*. Int J. Agric. Biol. 8, 618–620.

[B42] Jha A. B. Gali K. K. Tar'an B. Warkentin T. D. (2017). Fine mapping of QTLs for ascochyta blight resistance in pea using heterogeneous inbred families. Front. Plant Sci. 8:765. 10.3389/fpls.2017.00765 28536597 PMC5422545

[B43] Jha A. B. Tar'an B. Stonehouse R. Warkentin T. D. (2016). Identification of QTLs associated with improved resistance to ascochyta blight in an interspecific pea recombinant inbred line population. Crop Sci. 56, 2926–2939. 10.2135/cropsci2016.01.0001

[B44] Kaiser W. J. (1997). Inter- and intranational spread of ascochyta pathogens of chickpea, faba bean, and lentil. Can. J. Plant Pathol. 19, 215–224. 10.1080/07060669709500556

[B45] Kaiser W. J. Hannan R. M. (1986). Incidence of seedborne *Ascochyta lentis* in lentil germplasm. Phytopathology 76, 355–360. 10.1094/Phyto-76-355

[B46] Kaiser W. J. Wang B. C. Rogers J. D. (1997). *Ascochyta fabae* and *A. lentis:* host specificity, teleomorphs (Didymella), hybrid analysis and taxonomic status. Plant Dis. 81, 809–816. 10.1094/PDIS.1997.81.7.809 30861899

[B47] Kaur S. Cogan N. O. I. Pembleton L. W. Shinozuka M. Savin K. W. Materne M.. (2011). Transcriptome sequencing of lentil based on second-generation technology permits large-scale unigene assembly and SSR marker discovery. BMC Genomics 12:265. 10.1186/1471-2164-12-265 21609489 PMC3113791

[B48] Kaur S. Cogan N. O. I. Stephens A. Noy D. Butsch M. Forster J.. (2014a). EST-SNP discovery and dense genetic mapping in lentil (*Lens culinaris* Medik.) enable candidate gene selection for boron tolerance. Theor. Appl. Genet. 127, 703–713. 10.1007/s00122-013-2252-0 24370962

[B49] Kaur S. Kimber R. B. E. Cogan N. O. I. Materne M. Forster J. W. Paull J. G. (2014b). SNP discovery and high-density genetic mapping in faba bean (*Vicia faba*L.) permits identification of QTLs for ascochyta blight resistance. Plant Sci. 217–218, 47–55 10.1016/j.plantsci.2013.11.014 24467895

[B50] Khazaei H. Caron C. T. Fedoruk M. Diapari M. Vandenberg A. Coyne C. J. (2016). Genetic diversity of cultivated lentil (*Lens culinaris* Medik.) and its relation to the world's agro-ecological zones. Front. Plant Sci. 6:1093 10.3389/fpls.2016.01093 PMC4960256 27507980

[B51] Kimber R. B. E. Davidson J. A. Blake S. N. Russ M. H. Paull J. G. (2016). Virulence dynamics within *Ascochyta fabae* populations in Australia, in 4th International Ascochyta Workshop, (Troia), 46.

[B52] Kraft J. M. Dunne B. Goulden D. Armstrong S. (1998). A search for resistance in peas to *Ascochyta pinodes*. Plant Dis. 82, 251–253. 10.1094/PDIS.1998.82.2.251 30856811

[B53] Kumar S. K. Barpete S. Kumar J. Gupta P. Sarker A. (2013). Global lentil production: constraints and strategies. SATSA Mukhapatra Annu. Tech. Issue 17, 1–13.

[B54] Kumar S. Rajendran K. Kumar J. Hamwieh A. Baum M. (2015). Current knowledge in lentil genomics and its application for crop improvement. Front. Plant Sci. 6:78. 10.3389/fpls.2015.00078 25755659 PMC4337236

[B55] Lichtenzveig J. Bonfil D. J. Zhang H. B. Shtienberg D. Abbo S. (2006). Mapping quantitative trait loci in chickpea associated with time to flowering and resistance to *Didymella rabiei* the causal agent of Ascochyta blight. Theor. Appl. Genet. 113, 1357–1369. 10.1007/s00122-006-0390-3 17016689

[B56] Meuwissen T. H. E. Hayes B. J. Goddard M. E. (2001). Prediction of total genetic value using genome-wide dense marker maps. Genetics 157, 1819–1829. 11290733 10.1093/genetics/157.4.1819 PMC1461589

[B57] Moore K. Hobson K. Dron N. Harden S. Bithell S. Sambasivam P. (2016). Available online at: https://grdc.com.au/resources-and-publications/grdc-update-papers/tab-content/grdc-update-papers/2016/03/chickpea-ascochyta-latest-research-on-variability-and-implications-for-management

[B58] Morrall R. A. A. (1997). Evolution of lentil diseases over 25 years in western Canada. Can. J Plant. Pathol. 19, 197–207. 10.1080/07060669709500554

[B59] Morrall R. A. A. Sheppard J. W. (1981). Ascochyta blight of lentils in western Canada: 1978–1980. Can. Plant Dis. Surv. 61, 7–13.

[B60] Mustafa B. M. Coram T. E. Pang E. C. K. Taylor P. W. J. Ford R. (2009). A cDNA microarray approach to decipher lentil (*Lens culinaris*) responses to *Ascochyta lentis*. Australas. Plant Pathol. 38, 617–631. 10.1071/AP09048

[B61] Nasir M. Bretag T. W. (1997a). Pathogenic variability in Australian isolates of *Ascochyta lentis*. Australas. Plant Pathol. 26, 217–220. 10.1071/AP97036

[B62] Nasir M. Bretag T. W. (1997b). Prevalence of *Ascochyta fabae* f. sp. lentis on lentils seed from Victoria, Australia. Australas. Plant Pathol. 26, 117–120. 10.1071/AP97018

[B63] Nasir M. (1998). Improvement of Drought and Disease Resistance in Lentil in Nepal, Pakistan and Australia. Mid-term Report 1995–1998, Victorian Institute for Dryland Agriculture, Agriculture Victoria, Horsham.

[B64] Nasir M. Bretag T. W. (1998). Reactions of lentil accessions from 25 different countries to Australian isolates of *Ascochyta lentis*. Genet. Resour. Crop Evol. 45, 297–299. 10.1023/A:1008686007196

[B65] Newell M. A. Jannink J. L. (2014). Genomic selection in plant breeding. Methods Mol. Biol. 1145, 117–130. 10.1007/978-1-4939-0446-4_10 24816664

[B66] Nguyen T. Taylor P. Brouwer J. Pang E. Ford R. (2001). A novel source of resistance in lentil (*Lens culinaris* ssp. culinaris) to ascochyta blight caused by ascochyta lentis. Australas. Plant Pathol. 30, 211–215. 10.1071/AP01021

[B67] Pederson E. A. Morrall R. A. A. (1994). Effects of cultivar, leaf wetness duration, temperature and growth stage on infection and development of ascochyta blight on lentil. Phytopathology 84, 1024–1030. 10.1094/Phyto-84-1024

[B68] Pederson E. A. Morrall R. A. A. McCartney H. A. Fitt B. D. L. (1994). Dispersal of conidia of *Ascochyta fabae* f. sp. lentis from infected lentil plants by simulated wind and rain. Plant Pathol. 43, 50–55. 10.1111/j.1365-3059.1994.tb00552.x

[B69] Peever T. L. Barve M. P. Stone L. J. Kaiser W. J. (2007). Evolutionary relationships among *Ascochyta* species infecting wild and cultivated hosts in the legume tribes *Cicereae* and *Vicieae*. Mycologia 99, 59–77. 10.1080/15572536.2007.11832601 17663124

[B70] Prioul S. Frankewitz A. Deniot G. Morin G. Baranger A. (2004). Mapping of quantitative trait loci for partial resistance to *Ascochyta pinodes* in pea (*Pisum sativum* L.) at the seedling and adult plant stages. Theor. Appl. Genet. 108, 1322–1334. 10.1007/s00122-003-1543-2 14968300

[B71] Román B. Satovic Z. Avila C. M. Rubiales D. Moreno M. T. Torres A. M. (2003). Locating genes associated with Ascochyta fabae resistance in *Vicia faba*. Aust. J. Agric. Res. 54, 85–90. 10.1071/AR02034

[B72] Roundhill S. J. Fineran B. A. Cole A. L. J. Ingerfield M. (1995). Structural aspects of Ascochyta blight of lentil. Can. J. Bot. 73, 485–497. 10.1139/b95-049

[B73] Rubeena A. Taylor P. W. J. Ades P. K. Ford R. (2006). QTL mapping of resistance in lentil (*Lens culinaris* ssp. culinaris) to ascochyta blight (*Ascochyta lentis*). Plant Breed. 125, 506–512. 10.1111/j.1439-0523.2006.01259.x

[B74] Rubeena Ford R. Taylor P. W. J. (2003). Construction of an intraspecific linkage map of lentil (*Lens culinaris* ssp. culinaris). Theor. Appl. Genet. 107, 910–916. 10.1007/s00122-003-1326-9 12830386

[B75] Rubiales D. Trapero-Casas A. (2002). Occurrence of *Didymella fabae*, the teleomorph of *Ascochyta fabae*, on faba bean straw in Spain. J. Phytopathol. 150, 146–148. 10.1046/j.1439-0434.2002.00727.x

[B76] Sabbavarapu M. M. Sharma M. Chamarthi S. K. Swapna N. Rathore A. Thudi M. (2013). Molecular mapping of QTLs for resistance to *Fusarium wilt* (race 1) and Ascochyta blight in chickpea (*Cicer arietinum* L.). Euphytica 193, 121–133. 10.1007/s10681-013-0959-2

[B77] Sakr B. (1994). Inheritance and Linkage of Genetic Markers and Resistance to Ascochyta Blight in Lentil. Ph.D. thesis, Washington State University, Washington, DC.

[B78] Sambasivam P. Taylor P. W. J. Ford R. (2017). Pathogenic variation and virulence related responses of *Ascochyta lentis* on lentil. Eur. J. Plant Pathol. 147, 265–277. 10.1007/s10658-016-0999-2

[B79] Sharpe A. G. Ramsay L. Sanderson L. A. Fedoruk M. J. Clarke W. E. Rong L.. (2013). Ancient orphan crop joins modern era: gene-based SNP discovery and mapping in lentil. BMC Genomics 14:192. 10.1186/1471-2164-14-192 23506258 PMC3635939

[B80] Skiba B. Pang E. C. K. (2003). Mating trials and genetic study of virulence in *Ascochyta lentis* to the lentil cultivar ‘Northfield’. Aust. J. Agric. Res. 54, 453–460. 10.1071/AR02165

[B81] Sudheesh S. Rodda M. S. Davidson J. Javid M. Stephens A. Slater A. T.. (2016). SNP-based linkage mapping for validation of QTLs for resistance to ascochyta blight in lentil. Front. Plant Sci. 7:1604. 10.3389/fpls.2016.01604 27853461 PMC5091049

[B82] Sudheesh S. Rodda M. Kennedy P. Verma P. Leonforte A. Cogan N. O. I. (2015a). Construction of an integrated linkage map and trait dissection for bacterial blight resistance in field pea (*Pisum sativum* L.) *Mol. Breed* 35, 185 10.1007/s11032-015-0376-4

[B83] Sudheesh S. Sawbridge T. I. Cogan N. O. I. Kennedy P. Forster J. W. Kaur S. (2015b). De novo assembly and characterisation of the field pea transcriptome using RNA-Seq. BMC Genomics 16:611. 10.1186/s12864-015-1815-7 26275991 PMC4537571

[B84] Tar'an B. Buchwaldt L. Breitkreutz C. Tullu A. Warkentin T. Banniza S. (2002). Genetic study of Ascochyta blight resistance in chickpea and lentil. Available online at: http://www.usask.ca/soilsncrops/conference-proceedings/previous_years/Files/2002/2002_DOCS/Tar%27an_2002.pdf

[B85] Tar'an B. Buchwaldt L. Tullu A. Banniza S. Warkentin T. Vandenberg A. (2003b). Using molecular markers to pyramid genes for resistance to ascochyta blight and anthracnose in lentil (*Lens culinaris* Medik). Euphytica 134, 223–230. 10.1023/B:EUPH.0000003913.39616.fd

[B86] Tar'an B. Warkentin T. D. Tullu A. Vandenberg A. (2007). Genetic mapping of ascochyta blight resistance in chickpea (*Cicer arietinum* L.) using a simple sequence repeat linkage map. Genome 50, 26–34. 10.1139/g06-137 17546068

[B87] Tar'an B. Warkentin T. Somers D. J. Miranda D. Vandenberg A. Balde S. (2003a). Quantitative trait loci for lodging resistance, plant height and partial resistance to ascochyta blight in field pea (*Pisum sativum* L.). Theor. Appl. Genet. 107, 1482–1491. 10.1007/s00122-003-1379-9 12920512

[B88] Tay J. (1989). Inheritance of Resistance to Ascochyta Blight in Lentil. M.Sc. thesis, University of Saskatchewan, Saskatoon, SK.

[B89] Tay J. Slinkard A. (1989). Transgressive segregation for ascochyta resistance in lentil. Can. J. Plant Sci. 69:547.

[B90] Timmerman-Vaughan G. M. Frew T. J. Butler R. Murray S. Gilpin M. Falloon K.. (2004). Validation of quantitative trait loci for Ascochyta blight resistance in pea (*Pisum sativum* L.), using populations from two crosses. Theor. Appl. Genet. 109, 1620–1631. 10.1007/s00122-004-1779-5 15372153

[B91] Timmerman-Vaughan G. M. Frew T. J. Russell A. C. Khan T. Butler R. Gilpin M. (2002). QTL mapping of partial resistance to field epidemics of ascochyta blight of pea. Crop Sci. 42, 2100–2111. 10.2135/cropsci2002.2100

[B92] Tullu A. Banniza S. Tar'an B. Warkentin T. Vandenberg A. (2010). Sources of resistance to ascochyta blight in wild species of lentil (*Lens culinaris* Medik.). Genet. Resour. Crop Evol. 57, 1053–1063. 10.1007/s10722-010-9547-7

[B93] Tullu A. Tar'an B. Breitkreutz C. Banniza S. Warkentin T. Vandenberg A. (2006). A quantitative-trait locus for resistance to ascochyta blight (*Ascochyta lentis*) maps close to a gene for resistance to anthracnose (*Colletotrichum truncatum*) in lentil. Can. J. Plant. Pathol. 28, 588–595. 10.1080/07060660609507337

[B94] Udupa S. M. Baum M. (2003). Genetic dissection of pathotype-specific resistance to ascochyta blight disease in chickpea (*Cicer arietinum* L.) using microsatellite markers. Theor. Appl. Genet. 106, 1196–1202. 10.1007/s00122-002-1168-x 12748770

[B95] Vaghefi N. Mustafa B. M. Dulal N. Selby-Pham J. Taylor P. W. J. Ford R. (2013). A novel pathogenesis-related protein (LcPR4a) from lentil, and its involvement in defence against *Ascochyta lentis*. Phytopathol. Med. 52, 192–201. 10.14601/Phytopathol_Mediterr-11791

[B96] Vakulabharanam V. Slinkard A. Vandenberg A. (1997). Inheritance of resistance to ascochyta blight in lentil and linkage between isozyme, in Food Legume Research Conference III. (Adelaide, SA), 162.

[B97] Verma P. Goyal R. Chahota R. K. Sharma T. R. Abdin M. Z. Bhatia S. (2015). Construction of a genetic linkage map and identification of QTLs for seed weight and seed size traits in lentil (*Lens culinaris* Medik.). PLoS ONE 10:e0139666. 10.1371/journal.pone.0139666 26436554 PMC4593543

[B98] Xu Y. Crouch J. H. (2008). Marker-assisted selection in plant breeding: from publications to practice. Crop Sci. 48, 391–407 10.2135/cropsci2007.04.0191

[B99] Ye G. McNeil D. L. Hill G. D. (2002). Breeding for resistance to lentil ascochyta blight. Plant Breed. 121, 185–191. 10.1046/j.1439-0523.2002.00705.x

[B100] Zhang R. Hwang S. F. Chang K. F. Gossen B. D. Strelkov S. E. Turnbull G. D. (2006). Genetic resistance to *Ascochyta pinodes* in 558 field pea accessions. Crop Sci. 46, 2409–2414. 10.2135/cropsci2006.02.0089

[B101] Zohary D. (1999). Monophyletic vs. polyphyletic origin of the crops on which agriculture was founded in the Near East. Genet. Resour. Crop Evol. 46, 133–142. 10.1023/A:1008692912820

